# Similar Genetic Architecture of Alzheimer’s Disease and Differential *APOE* Effect Between Sexes

**DOI:** 10.3389/fnagi.2021.674318

**Published:** 2021-05-28

**Authors:** Hao Wang, Min-Tzu Lo, Sara Brin Rosenthal, Carolina Makowski, Ole A. Andreassen, Rany M. Salem, Linda K. McEvoy, Mark Fiecas, Chi-Hua Chen

**Affiliations:** ^1^Department of Radiology, Center for Multimodal Imaging and Genetics, University of California, San Diego, San Diego, CA, United States; ^2^Center for Computational Biology and Bioinformatics, University of California, San Diego, San Diego, CA, United States; ^3^Division of Mental Health and Addiction, NORMENT Centre, University of Oslo and Oslo University Hospital, Oslo, Norway; ^4^Department of Family Medicine and Public Health, Division of Epidemiology, University of California, San Diego, San Diego, CA, United States; ^5^Herbert Wertheim School of Public Health and Human Longevity Science, University of California, San Diego, San Diego, CA, United States; ^6^School of Public Health, Division of Biostatistics, University of Minnesota, Minneapolis, MN, United States

**Keywords:** Alzheimer’s disease, sex difference, heritability, genetic heterogeneity, genome-wide association study

## Abstract

Sex differences have been observed in the clinical manifestations of Alzheimer’s disease (AD) and elucidating their genetic basis is an active research topic. Based on autosomal genotype data of 7,216 men and 10,680 women, including 8,136 AD cases and 9,760 controls, we explored sex-related genetic heterogeneity in AD by investigating SNP heritability, genetic correlation, as well as SNP- and gene-based genome-wide analyses. We found similar SNP heritability (men: 19.5%; women: 21.5%) and high genetic correlation (*R*_g_ = 0.96) between the sexes. The heritability of *APOE* ε4-related risks for AD, after accounting for effects of all SNPs excluding chromosome 19, was nominally, but not significantly, higher in women (10.6%) than men (9.7%). In age-stratified analyses, ε3/ε4 was associated with a higher risk of AD among women than men aged 65–75 years, but not in the full sample. Apart from *APOE*, no new significant locus was identified in sex-stratified gene-based analyses. Our result of the high genetic correlation indicates overall similar genetic architecture of AD in both sexes at the genome-wide averaged level. Our study suggests that clinically observed sex differences may arise from sex-specific variants with small effects or more complicated mechanisms involving epigenetic alterations, sex chromosomes, or gene-environment interactions.

## Introduction

Alzheimer’s disease (AD) is a neurodegenerative disorder characterized by progressive memory loss and is the most common form of dementia (Winblad et al., 2016). The majority of cases are the sporadic form of late-onset AD. The estimated prevalence of AD among adults over the age of 65 years in the United States is about 10%, and approximately two-thirds of AD patients are women (Alzheimer’s Association, 2020).

Phenotypic differences between sexes in AD have long been noted in various aspects (Dubal, 2020). For example, the cognitive and psychiatric symptoms present differently between women and men (Mielke et al., 2014; Snyder et al., 2016; Pike, 2017; Ferretti et al., 2018; Laws et al., 2018). Longitudinal data suggest greater cognitive decline and hippocampal atrophy rates in women after diagnosis of mild cognitive impairment (MCI) or AD (Hua et al., 2010; Holland et al., 2013; Lin et al., 2015; Gamberger et al., 2017), even though few differences have been reported to date in biomarkers of AD, such as accumulation of amyloid-β and tau proteins (Ferretti et al., 2018). Epidemiologically, the risk and protective factors of AD distribute unequally between the sexes. For instance, there is a higher prevalence of risk factors in postmenopausal women, such as cardiovascular diseases, depression, and sleep disorders; whereas educational attainment and physical activity tend to be higher in men, which are protective factors of AD (Xu et al., 2016; Stephen et al., 2017). In addition, evidence suggests that there are risk factors specific to women, including factors related to reproductive history, pregnancy complications, or hormone replacement therapy (Ferretti et al., 2018; Nebel et al., 2018; Gilsanz et al., 2019). In general, the lifetime risk of developing AD in those aged 65 years or older is twice as high in women than in men (21.2% vs. 11.6%, respectively; Alzheimer’s Association, 2020). However, the reasons for these sex differences are not completely clear to date.

As a complex polygenic disease, the etiology of AD may reflect a combination of genetic and environmental effects. In this study, we specifically focused on the genetic architecture that characterizes the genetic factors underlying this heterogeneity in the context of a polygenic framework, considering the collective effects of multiple genetic risk variants (Timpson et al., 2018). Emerging evidence suggests that the effect of the apolipoprotein E (*APOE*) ε4 allele, a major genetic risk factor for AD, is modified by sex (Farrer et al., 1997; Altmann et al., 2014). Notably, a large-scale meta-analysis demonstrated that although the *APOE* ε4 allele confers generally a similar risk of developing AD in women and men aged 55–85 years, noteworthy differences can be found when stratifying patients by age groups (Neu et al., 2017). Specifically, this study found that ε3/ε4 was associated with an increased risk of AD in women compared to men between the ages of 65–75 years. It has also been supported by neuroimaging studies which demonstrated significant *APOE*-by-sex interaction in the distribution of cerebral hypometabolism and changes in cortical thickness (Sampedro et al., 2015), as well as a higher prevalence of *APOE*-ε4-associated cerebral small vessel disease in male AD patients (Finch and Shams, 2016).

Previous studies revealed multiple variants with significant sex-by-genotype interactions in AD (Dumitrescu et al., 2019; Gamache et al., 2020), and genome-wide association studies (GWAS) have identified many AD susceptibility loci in addition to *APOE* (Harold et al., 2009; Lambert et al., 2009; Seshadri et al., 2010; Hollingworth et al., 2011; Naj et al., 2011; Jansen et al., 2019; Kunkle et al., 2019). However, due to low statistical power to robustly detect sex-specific loci in AD after sample stratification, few studies have investigated sex difference effects through a classical GWAS approach to identify variants associated with the AD diagnosis in case-control cohorts (Nazarian et al., 2019). Other study paradigms such as incorporating family-based association design or leveraging neuropathological features as AD endophenotypes have found sex-specific associations (Deming et al., 2018; Dumitrescu et al., 2019; Prokopenko et al., 2020). In addition to detecting sex-specific loci, some studies identified significant sex-specific predictors for AD phenotypes such as neuropathology (Deming et al., 2018), biomarkers (Dumitrescu et al., 2019), and age at onset using polygenic hazard scores (Fan et al., 2020). In the present study, we estimated sex-stratified single nucleotide polymorphism (SNP) heritability of AD and genetic correlation between sexes, in which a large number of common SNPs with small effects contribute additively to phenotypic variation. Although this polygenic model cannot detect sex-specific loci, it allows us to investigate the genetic architecture of AD between sexes with adequate statistical power using the Alzheimer’s Disease Genetics Consortium (ADGC) sample. We then examined *APOE* heritability and the odds ratio (OR) for AD with mixed linear models, and lastly performed exploratory sex-stratified genome-wide analyses.

## Materials and Methods

### ADGC Sample

The 2-phase ADGC data include 15 cohorts in both phases with 18,844 and 5,342 individuals, respectively, of European ancestry aged 60 years and above (except 1 AD patient with the age at onset at 58 years old), who were enrolled between 1989 and 2011. The ADGC data also include common covariates (age at onset of AD or age at the first visit for controls, sex, and top 10 principal components) to correct for population stratification. The details of each cohort in phase 1 and phase 2 are shown in Supplementary Table 1. Quality control was conducted on genotyping call rate, X-chromosome analysis for sex, and identity by descent for relatedness and sample duplication (Jun et al., 2010; Naj et al., 2011). Genotyped SNPs with low minor allele frequencies (<0.02 for Affymetrix chips or <0.01 for Illumina chips) or violation of Hardy-Weinberg equilibrium (*P* value < 10^−6^) were excluded. Genome-wide SNP imputation was performed in each cohort using the 1,000 Genomes reference panel and imputed SNPs were removed if imputation *quality* (*R*^2^) < 0.5 (Jun et al., 2010).

### Whole-Genome SNP Heritability and Genetic Correlation Estimation

The sex-stratified SNP heritability estimates of AD were calculated as the proportion of phenotypic variance explained by SNPs from the whole genome, implemented by Genome-wide Complex Trait Analysis (GCTA; Yang et al., 2011a). GCTA fits effects of all SNPs simultaneously as random effects and effects of other covariates (age, cohort indicators, and the top 10 principal components) as fixed effects in a mixed linear model. In the regression model, the variance explained by SNPs can be estimated by the restricted maximum likelihood (REML) approach using the genetic relationship matrix (GRM), which reflects the genetic correlations between individuals (Yang et al., 2010). In our analysis, SNPs with minor allele frequencies >0.01 were retained to estimate the GRM, and related individuals were excluded if individual-pairwise GRM >0.025. The SNP heritability estimates were also partitioned through two independent GRMs into chromosome 19, which harbors the *APOE* region, and the remaining 21 chromosomes (Yang et al., 2011b). The genetic correlation between sexes was estimated using the bivariate REML method (Lee et al., 2012), which implies genetic heterogeneity if it significantly differs from 1.

A total of 7,216 males and 10,680 females were included for both analyses combining cohorts of both ADGC phase 1 and 2, and the statistical power of the genetic correlation analysis was evaluated using the GCTA-GREML power calculator^1^. With the above sample sizes, estimated disease prevalence in the population, the lowest estimated SNP heritability of 0.19 as previously reported (Zhang et al., 2020), type I error rate (α) of 0.05 and the default variance explained by SNP-derived genetic relationships of 2 × 10^−5^, the calculated power was 1.0 for both sex-stratified analyses.

We used the AD prevalence estimates to correct ascertainment bias due to oversampled cases in case-control study studies (Lee et al., 2011). As AD accounts for the majority of dementia cases, we estimated AD prevalence by using age-and gender-specific estimates of dementia prevalence in the United States from a systemic meta-analysis, which included 5-year prevalence for those over 60 years of age in males and females (Prince et al., 2013). We re-calculated average prevalence for males and females (Supplementary Table 2) weighted by age-and sex-specific annual estimates of the resident population of the United States in 2015 from the United States Census Bureau^2^ (Lee et al., 2011). This resulted in a prevalence of 0.055 in males and 0.072 in females. The resulting prevalence information was only used in the GCTA analyses above.

### *APOE* ε4 SNP Heritability Estimation

To estimate heritability attributable to the *APOE* ε4 alleles, we included only the GRM generated from all chromosomes excluding chromosome 19, including the same covariates in the mixed linear model as above. We then calculated the best linear unbiased prediction (BLUP), which is the total genetic effect and residual effect for each individual (Yang et al., 2011a). We regressed residuals generated from BLUP estimation in a linear model on the number of *APOE* ε4 alleles and obtained *R*^2^ for males (*N* = 6,896) and females (*N* = 10,150) separately, which is the proportion of the variance of the residuals explained by *APOE* ε4 alleles and denotes the heritability of *APOE* ε4 alleles.

The effect sizes of one and two *APOE* ε4 alleles were also estimated by calculating the ORs between AD and control groups in the logistic regression model. In addition, we studied the ORs in younger and older age groups with a cut-off of 80 years old, which was selected based on our prior analysis that indicated a greater genetic heterogeneity between these age groups, and previous studies that suggested a reduced risk of AD associated with ε4 among the population above 80 years old (Bonham et al., 2016; Neu et al., 2017; Lo et al., 2019a), although data are needed to replicate the results. We also specifically compared the ORs of ε3/ε4 and ε3/ε3 in participants ages 65–75 years, based on the previous publication that reported a higher risk conferred by ε3/ε4 in women than in men in this age group (Neu et al., 2017).

The linear and logistic modeling were computed in R, and the confidence intervals of *R*^2^ were calculated using the CI.Rsq function in the psychometric package for R. The sample sizes were slightly smaller due to missing *APOE* ε4 status for some individuals.

### Exploratory SNP-Based GWAS of AD

GWAS of 38,043,082 SNPs were separately performed in males and females using logistic regressions implemented in PLINK 1.9 (Chang et al., 2015). Age at disease onset of AD (or age at the first visit for the control group), cohort indicators, and the top 10 principal components were included as covariates. Subjects with individual-pairwise GRM > 0.1 were excluded from analyses to ensure sample independence (Wray et al., 2013). A total of 8,682 males (4,010 cases and 4,672 controls) and 12,772 females (5,705 cases and 7,067 controls) were included combining cohorts of both ADGC phase 1 and 2. Significant SNPs with genome-wide *p*-value < 5 × 10^−8^ were obtained, and clumped using the European reference panel of the 1,000 Genomes Project phase 3 (released in May 2013; Auton et al., 2015), to remove correlated SNPs with LD *r*^2^ > 0.1 within 250 kb of the top SNP using PLINK 1.9 to obtain LD-independent SNPs (Chang et al., 2015). We used the METAL software to implement Cochrane’s *Q* test for heterogeneity for each SNP between male and female GWAS (Willer et al., 2010).

### Gene-Based Analyses

To reduce the number of tests conducted in SNP-based GWAS and aggregate the small effect of each SNP within a gene, we performed sex-stratified gene-based analyses using MAGMA v1.08 implemented in FUMA v1.3.6a (Watanabe et al., 2017). The gene-based *p*-value was calculated based on the mean of the summary statistic (*χ*^2^ statistic) of GWAS for the SNPs in a gene (de Leeuw et al., 2015; Watanabe et al., 2017). SNPs with minor allele frequencies ≥0.01 in the European reference panel of 1,000 Genomes Project were included. The distance between two LD blocks < 250 kb was merged into a locus. In our analyses, SNPs within the genes were mapped to 18,338 loci (genes). The significant *p*-value was determined by the Bonferroni method, which divides 0.05 by the number of genes (19,151) resulting in 2.61 × 10^−6^. The sex-stratified gene-based analyses using summary statistics from sex-stratified GWAS were performed to obtain significant genes for males and females.

### Verification of Results With Matched Female Sub-cohort

As the sizable difference in sample sizes between the two sex strata led to discrepancy of statistical power (male-to-female ratio: 1:1.5) and might bias our analysis on sex difference, we formed a female sub-cohort with matched numbers of cases and controls as the male cohort by random selection, and repeated the age-and sex-stratified analyses on the *APOE*-ε4 effects, as well as the genome-wide SNP and gene-based analyses.

## Results

### Whole-Genome SNP Heritability and Genetic Correlation Estimates

The whole-genomic heritability estimates of AD were 19.5% (95% CI: 9.9–29.1%) in males and 21.5% (95% CI: 15.0–28.1%) in females respectively, and overall 20.6% (95% CI: 16.4–24.8%) among the combined ADGC phase 1 and 2 cohorts.

**Figure 1:**
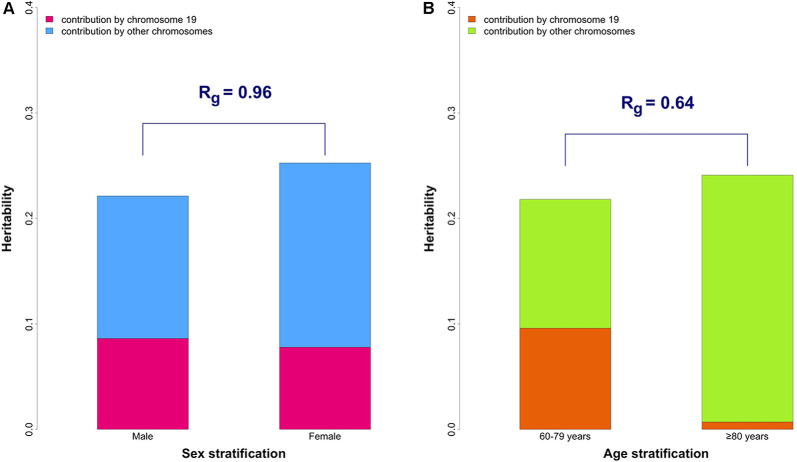
The overall and partitioned heritability estimates in combined phase 1 and 2 samples. **(A)** Comparison between sexes. The heritability estimates were 19.5% (95% CI: 9.9–29.1%) and 21.5% (95% CI: 15.0–28.1%) in male (*N* = 7,216) and female (*N* = 10,680) groups, respectively. Heritability estimates of chromosome 19 are 8.6% (95% CI: 5.9–11.4%) in males and 7.8% (95% CI: 5.8–9.8%) in females. The genetic correlation (R_g_) between the two sexes was 0.96 (*p*-value for H_0_: *R*_g_ = 1 was 0.42). **(B)** Comparison between younger (60–79 years old) and older (≥ 80 years old) groups (from our previously published data; Lo et al., 2019b). The genetic correlation (R_g_) between the two age groups is 0.64 which significantly differs from 1 (*p*-value for H_0_: *R*_g_ = 1 was 0.043).

The heritability estimates partitioned by chromosome 19 and other chromosomes are shown in Figure 1A and Supplementary Table 3. The contribution of chromosome 19 was similar in males and female, which is in contrast to the results that we previously reported in age-stratified analysis (Figure 1B).

The genetic correlation (R_g_) between males and females was 0.96 (*p*-value for H_0_: *R*_g_ = 1 was 0.42) for the whole genome. The results were unchanged after excluding chromosome 19 (*R*_g_ = 0.96, *p*-value for H_0_: *R*_g_ = 1 was 0.43), suggesting overall genetic homogeneity between sexes in AD.

### *APOE* ε4 SNP Heritability Estimation

The heritability of AD due to *APOE* ε4 was estimated to be 9.7% (95% CI: 8.4–11.0%) in males (*N* = 6,896), 10.6% (95% CI: 9.5–11.8%) in females (*N* = 10,150), and 10.2% (95% CI: 9.4–11.1%) in the whole sample.

*APOE* ε4-associated risk of AD was similar between males and females, with ORs of 3.85 (95% CI: 3.40–4.37) for AD in males and 4.10 (95% CI: 3.71–4.52) in females with one ε4 allele, and ORs of 13.24 (95% CI: 9.69–18.26) and 11.59 (95% CI: 9.19–14.76) in males and females with two ε4 alleles compared to non-carriers. Stratification by age-at-onset of AD demonstrated higher *APOE* ε4-associated ORs in the younger group (onset at 60–80 years old) compared to the older group (onset later than 80 years old) in both males and females as shown in Supplementary Table 4, suggesting a higher genetic risk conferred by *APOE* ε4 alleles among younger patients. Consistent results were seen in the female sub-cohort with matched case and control numbers as the male cohort (Supplementary Table 5).

Although we did not observe significant differences between men and women in *APOE* ε4-associated ORs in this age group, the subgroup analysis comparing ε3/ε4 and ε3/ε3 among the narrower age group of 65–75 years demonstrated a higher risk in females (OR: 5.93, 95% CI: 4.88–7.22) than males (OR: 3.51, 95% CI: 2.78–4.44), which was also observed in the female sub-cohort (OR: 5.92, 95% CI: 4.68–7.51). No notable sex differences were found in the other age or *APOE* genotype subgroups.

### SNP-Based GWAS of AD

Significant SNPs in the *APOE* region and *BIN1* were identified, which have been reported in previous GWAS. No novel SNP was detected in either sex from SNP-based GWAS, and no genome-wide significant (*p* < 5 × 10^−8^ in GWAS) LD-independent SNPs with significantly different effect sizes between sexes (heterogeneity *p* < 0.05) were identified by heterogeneity Cochrane’s *Q* tests (Supplementary Table 6).

### Gene-Based Analyses

**Figure 2:**
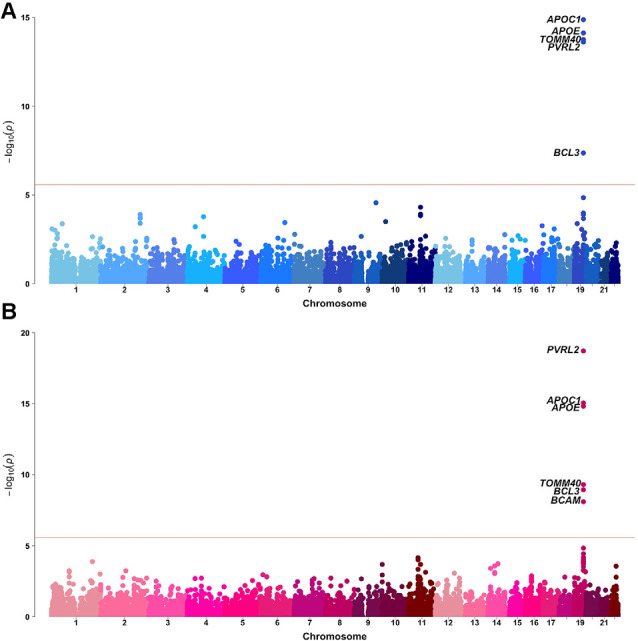
Manhattan plots of MAGMA-annotated genes in Alzheimer’s disease genetics consortium (ADGC) combined phase 1 and phase 2 samples of **(A)** male and **(B)** female strata. The red line denotes the gene-based genome-wide significance level of *P*-value = 2.61 × 10^−6^.

Sex-stratified gene-based analyses were then performed in 8,682 males and 12,772 females. Apart from *APOE, APOC1, TOMM40, PVRL2, BCL3, and BCAM* on chromosome 19, no novel genome-wide significant gene was identified (Figure 2, Table 1). *BCAM* was significant among females only, but the Cochrane’s *Q* test demonstrated no sex-related heterogeneity for the SNPs within this gene (range of heterogeneity *p*-values: 0.27–0.91). In addition, *BCAM* was not significant in the female sub-cohort (Supplementary Figure 1, Supplementary Table 7), consistent with the result from the heterogeneity *Q* test using the full sample showing no sex-related effect.

**Table 1:** Significant MAGMA-annotated genes (*p*-value < 2.61 × 10^−6^) based on sex-stratified GWAS.

					Male				Female				Heterogeneity between sexes
Gene	Chr	*p*-value	Top SNP in Gene	A1/A2	Freq	N	OR (95% CI)	*p*-value	Freq	N	OR (95% CI)	*p*-value	*p*-value
**Top genes in male**													
*APOC1*	19	1.33 × 10^−15^	rs12721051	G/C	0.755	7,374	2.80 (2.70–2.90)	9.71 × 10^−97^	0.761	10,968	3.16 (2.37–3.94)	1.89 × 10^−182^	0.060
*APOE*	19	7.49 × 10^−15^	rs429358	C/T	0.774	8,060	3.25 (3.15–3.34)	5.04 × 10^−122^	0.784	11,793	3.66 (3.57–3.74)	1.78 × 10^−215^	0.067
*TOMM40*	19	1.77 × 10^−14^	rs59007384	T/G	0.713	7,699	2.42 (2.33–2.51)	7.85 × 10^−85^	0.720	11,249	2.49 (2.42–2.56)	1.20 × 10^−138^	0.614
*PVRL2*	19	2.42 × 10^−14^	rs6857	T/C	0.762	7,955	2.81 (2.71–2.90)	4.65 × 10^−101^	0.769	11,613	2.97 (2.89–3.05)	1.95 × 10^−171^	0.367
*BCL3*	19	4.30 × 10^−8^	rs2965169	C/A	0.610	6,932	0.81 (0.73–0.90)	5.80 × 10^−7^	0.606	10,174	0.82 (0.76–0.89)	2.12 × 10^−9^	0.832
**Top genes in female**													
*PVRL2*	19	1.93 × 10^−19^	rs6857	T/C	0.762	7,955	2.81 (2.71–2.90)	4.65 × 10^−101^	0.769	11,613	2.97 (2.89–3.05)	1.95 × 10^−171^	0.367
*APOC1*	19	8.88 × 10^−16^	rs12721051	G/C	0.755	7,374	2.80 (2.70–2.90)	9.71 × 10^−97^	0.761	10,968	3.16 (2.37–3.94)	1.89 × 10^−182^	0.060
*APOE*	19	1.50 × 10^−15^	rs429358	C/T	0.774	8,060	3.25 (3.15–3.34)	5.04 × 10^−122^	0.784	11,793	3.66 (3.57–3.74)	1.78 × 10^−215^	0.067
*TOMM40*	19	5.00 × 10^−10^	rs59007384	T/G	0.713	7,699	2.42 (2.33–2.51)	7.85 × 10^−85^	0.720	11,249	2.49 (2.42–2.56)	1.20 × 10^−138^	0.614
*BCL3*	19	1.15 × 10^−9^	rs2965169	C/A	0.610	6,932	0.81 (0.73–0.90)	5.80 × 10^−7^	0.606	10,174	0.82 (0.76–0.89)	2.12 × 10^−9^	0.832
*BCAM*	19	8.00 × 10^−9^	rs28399637	A/G	0.714	5,028	1.52 (1.41–1.62)	1.69 × 10^−15^	0.719	7,329	1.54 (1.45- 1.62)	3.71 × 10^−25^	0.852

*The top SNPs with smallest p-values within genes are shown. Abbreviations: Chr, chromosome; A1, effect allele; A2, non-effect allele; Freq, allele frequency of A1; N, sample size; OR, odds ratio; CI, confidence interval*.

## Discussion

The present study demonstrated a largely similar genetic basis of AD between males and females. Results of partitioned SNP heritability showed similar genetic effects of both the *APOE*-harboring chromosome 19 and the remaining 21 chromosomes in AD in males and females, as well as a high genetic correlation, which captures the genome-wide architecture of AD. These results indicate that the overall genetic underpinnings and architecture of AD are similar across sexes, in contrast to the genetic heterogeneity across age as identified previously (Lo et al., 2019a).

Targeted analyses on *APOE* ε4 alleles demonstrated no disparity in heritability of AD between sexes, but higher heritability in the early-onset groups of both sexes (Lo et al., 2019a). We did not find sex differences in *APOE* ε4 allele-associated genetic risk of AD using the entire age group or subgroups of 60–80 and >80 years old, but specific subgroup analysis replicated the previously reported finding that ε3/ε4 confers higher risk in women than men aged 65–75 years (Neu et al., 2017). We noted that this was not a completely independent replication because up to 58.5% of our samples (*n* = 21,454) overlap with 21.6% of the sample (*n* = 57,979) in the prior report (Neu et al., 2017). This may reflect intricate interactions between age, sex, and *APOE*-ε4, involving pleiotropy, tauopathy, and estrogen response of *APOE* (Riedel et al., 2016).

As a pilot study, the exploratory sex-stratified GWAS did not identify any new loci with significant sex-related heterogeneity. The *APOE-APOC1-TOMM40* region in chromosome 19 was significantly associated with AD in both sexes, although substantial sex-related changes in lipid metabolism may be associated with this region. Emerging data support the role of APOE lipidation and brain lipid transport in the development of AD (Husain et al., 2021). It is evident that estrogen regulates the expression and synthesis of APOE, and APOE facilitates the neuroprotective effects of estrogens and androgens, suggesting the sex hormone-APOE interaction may underlie the sex difference in AD (Gamache et al., 2020).

## Implications

As a multifactorial disease, sex-related phenotypic diversity in AD has been noted in multiple studies. The observed differences have been described to arise from combined effects of genetic, epigenetic, cellular and, environmental mechanisms leading to a heterogeneous disease etiology, especially for late-onset AD. In the present study, we found a similar genetic architecture of AD between women and men, which implies that effect sizes of sex-difference variants are likely to be small and detecting these variants through a classical GWAS approach requires a larger sample than the current one. It is likely that age-by-sex interactions in AD further complicate detecting sex-difference variants. Additionally, there is a likely crucial role for gene-environmental interaction at multiple epigenetic levels for the observed sex differences in AD (Guo et al., 2021). Further systematic studies on epigenomic, gene expression, and immunomic profiling, as well as the inclusion of a larger spectrum of environmental factors, may provide greater insight into the sex heterogeneity underlying AD.

## Limitations

The present study is limited by multiple factors. Although the GCTA power was adequate, the exploratory sex-stratified GWA studies were underpowered given the available sample size. There was also a sizable difference in sample sizes with a male-to-female ratio of close to 1:1.5, although we verified the results with a matched female sub-cohort to avoid false positive findings simply due to discrepancy in statistical power. In addition, effects of sex chromosomes were not included in this study, which may also be crucial in AD or aging (McCartney et al., 2019).

## Conclusion

In the present study, we discovered a high genetic correlation of AD between men and women. The overall genetic architecture of AD is similar between sexes, in contrast to genetic heterogeneity across age. Previously reported higher risk from *APOE* ε3/ε4 genotype in females than males among the age group 65–75 years was replicated. Effect sizes of sex-difference variants are likely to be small and large GWAS are needed for discovering such variants. Sex-specific effects from epigenetic variations and gene-environment interactions warrant future investigation to reveal the underlying mechanisms that explain the clinically observed sex differences in AD.

## Data Availability Statement

The data that support the findings of this study are available through the National Institute on Aging Genetics of Alzheimer’s Disease Data Storage Site (NIAGADS), NIA’s qualified access data repository (https://www.niagads.org/home).

## Author Contributions

C-HC, HW, and M-TL contributed to the conception and design of the study. HW, M-TL, RS, MF, and C-HC contributed to the data analysis. HW, M-TL, SR, CM, OA, RS, LM, MF, and C-HC contributed to data interpretation. HW, M-TL, LM, MF, and C-HC contributed to drafting the manuscript. All authors contributed to the article and approved the submitted version.

## Conflict of Interest

The authors declare that the research was conducted in the absence of any commercial or financial relationships that could be construed as a potential conflict of interest.

## Footnotes

^1^https://shiny.cnsgenomics.com/gctaPower

^2^https://www2.census.gov/programs-surveys/popest/datasets/2010-2015/national/asrh/nc-est2015-agesex-res.csv
