# Supplementary material for: Similar Genetic Architecture of Alzheimer’s Disease and Differential APOE Effect Between Sexes
Source: Front Aging Neurosci. 2021 May 28;13:674318. doi: 10.3389/fnagi.2021.674318 (PMC8194397; doi:10.3389/fnagi.2021.674318)
Supplement: Supplementary file 1 [file Data_Sheet_1.docx]

Supplementary Material

**Supplementary Table 1**. Sample sizes and sex/age distributions in each cohort for GWAS stratified by age 60-79 and ≥ 80 years.

|  |  | Total | |  |  | Age 60-79 years | | | | |  |  | Age ≥ 80 years | | | | |  |
| --- | --- | --- | --- | --- | --- | --- | --- | --- | --- | --- | --- | --- | --- | --- | --- | --- | --- | --- |
| Cohort | No. of indiv. | | Prop. of Female (%) | | No. of indiv. | | Prop. of Female (%) | Mean age | No. of cases | No. of controls | | No. of indiv. | | Prop. of Female (%) | Mean age | No. of cases | No. of controls | |
| **Phase 1** |  | |  | |  | |  |  |  |  | |  | |  |  |  |  | |
| ACT1 | 1866 | | 57.07 | | 680 | | 50.74 | 75.11 | 53 | 627 | | 1186 | | 60.71 | 85.20 | 258 | 928 | |
| ADC^a^ | 3598 | | 55.95 | | 2755 | | 53.83 | 70.36 | 2028 | 727 | | 843 | | 62.87 | 84.90 | 496 | 347 | |
| ADNI | 310 | | 42.90 | | 211 | | 45.50 | 72.64 | 107 | 104 | | 99 | | 37.37 | 83.60 | 31 | 68 | |
| GSK | 1351 | | 60.77 | | 1069 | | 61.46 | 71.93 | 520 | 549 | | 282 | | 58.16 | 83.68 | 127 | 155 | |
| MAYO | 1035 | | 51.21 | | 1028 | | 51.07 | 72.86 | 0 | 1028 | | 7 | | 71.43 | 80.00 | 0 | 7 | |
| MIRAGE | 631 | | 61.49 | | 546 | | 61.90 | 69.81 | 346 | 200 | | 85 | | 58.82 | 83.19 | 40 | 45 | |
| NIA-LOAD | 1605 | | 62.62 | | 1226 | | 63.62 | 71.38 | 594 | 632 | | 379 | | 59.37 | 84.41 | 130 | 249 | |
| OHSU | 276 | | 58.70 | | 47 | | 44.68 | 74.09 | 10 | 37 | | 229 | | 61.57 | 87.16 | 115 | 114 | |
| ROSMAP1 | 1019 | | 71.23 | | 292 | | 72.60 | 74.32 | 48 | 244 | | 727 | | 72.08 | 86.59 | 227 | 500 | |
| TGEN2 | 74 | | 43.24 | | 62 | | 41.94 | 71.57 | 62 | 0 | | 12 | | 50.00 | 84.88 | 12 | 0 | |
| UMV | 1950 | | 63.18 | | 1521 | | 62.79 | 70.53 | 750 | 771 | | 429 | | 64.57 | 84.08 | 239 | 190 | |
| UPITT | 1951 | | 63.25 | | 1568 | | 62.50 | 71.84 | 954 | 614 | | 383 | | 66.32 | 82.86 | 188 | 195 | |
| WASHU1 | 493 | | 57.40 | | 353 | | 57.79 | 71.02 | 231 | 122 | | 140 | | 56.43 | 85.16 | 79 | 61 | |
| **Total** | 16159 | | 59.62 | | 11358 | | 58.30 | 71.52 | 5703 | 5655 | | 4801 | | 62.74 | 84.94 | 1942 | 2859 | |
| **Phase 2** |  | |  | |  | |  |  |  |  | |  | |  |  |  |  | |
| ACT2 | 29 | | 62.07 | | 5 | | 60.00 | 72.00 | 4 | 1 | | 24 | | 62.50 | 85.88 | 17 | 7 | |
| ADC^a^ | 1990 | | 61.31 | | 1396 | | 59.67 | 70.80 | 627 | 769 | | 594 | | 65.15 | 85.37 | 156 | 438 | |
| BIOCARD | 118 | | 61.02 | | 111 | | 61.26 | 67.12 | 5 | 106 | | 7 | | 57.14 | 83.00 | 1 | 6 | |
| CHAP2 | 169 | | 54.44 | | 58 | | 53.45 | 74.72 | 9 | 49 | | 111 | | 54.96 | 86.29 | 18 | 93 | |
| EAS | 147 | | 42.18 | | 33 | | 42.42 | 77.09 | 1 | 32 | | 114 | | 42.11 | 85.95 | 5 | 109 | |
| MTV | 403 | | 59.06 | | 323 | | 58.20 | 69.88 | 173 | 150 | | 80 | | 62.50 | 83.28 | 44 | 36 | |
| NBB | 48 | | 56.25 | | 15 | | 46.67 | 70.07 | 0 | 15 | | 33 | | 60.61 | 86.73 | 0 | 33 | |
| RMAYO | 235 | | 40.00 | | 137 | | 43.80 | 75.18 | 2 | 135 | | 98 | | 34.69 | 84.89 | 6 | 92 | |
| ROSMAP2 | 183 | | 77.05 | | 77 | | 80.52 | 73.58 | 0 | 77 | | 106 | | 74.53 | 84.84 | 0 | 106 | |
| TARC1 | 498 | | 62.85 | | 381 | | 61.68 | 70.76 | 249 | 132 | | 117 | | 66.67 | 83.56 | 68 | 49 | |
| UKS | 765 | | 56.08 | | 676 | | 54.44 | 68.81 | 506 | 170 | | 89 | | 68.54 | 82.66 | 89 | 0 | |
| WASHU2 | 89 | | 55.06 | | 77 | | 55.84 | 69.71 | 16 | 61 | | 12 | | 50.00 | 83.00 | 2 | 10 | |
| WHICAP | 621 | | 61.77 | | 248 | | 56.05 | 75.10 | 21 | 227 | | 373 | | 65.42 | 86.42 | 51 | 322 | |
| **Total** | 5295 | | 59.26 | | 3537 | | 57.99 | 70.85 | 1613 | 1924 | | 1758 | | 61.83 | 85.28 | 457 | 1301 | |
| **Phase 1&2** |  | |  | |  | |  |  |  |  | |  | |  |  |  |  | |
| **All cohorts** | 21454 | | 59.53 | | 14895 | | 58.23 | 71.36 | 7316 | 7579 | | 6559 | | 62.49 | 85.03 | 2399 | 4160 | |

^a^We combined three cohorts (ADC1, ADC2, ADC3) in phase 1 (Jun et al., 2010; Naj et al., 2011) and three cohorts (ADC4, ADC5, ADC6) in phase 2 (Jun et al., 2016) for covariate adjustment, as source cohorts of individuals were recruited in the same center with identical criteria and quality control for phenotype and genotype data.

**Supplementary Table 2**. Age and sex-specific dementia prevalence and population.

| Age Group |  | Prevalence estimate^a^ | |  |  | Population | | |  |
| --- | --- | --- | --- | --- | --- | --- | --- | --- | --- |
|  | Male | | Female | | Total | | Male | Female | |
| 60-64 | 0.013 | | 0.010 | | 19,069,877 | | 9,117,180 | 9,952,697 | |
| 65-69 | 0.021 | | 0.018 | | 16,067,468 | | 7,596,190 | 8,471,278 | |
| 70-74 | 0.037 | | 0.033 | | 11,483,049 | | 5,296,158 | 6,186,891 | |
| 75-79 | 0.068 | | 0.064 | | 8,123,833 | | 3,610,906 | 4,512,927 | |
| 80-84 | 0.123 | | 0.125 | | 5,799,341 | | 2,412,665 | 3,386,676 | |
| 85-89 | 0.216 | | 0.232 | | 3,864,345 | | 1,442,244 | 2,422,101 | |
| ≥ 90 | 0.452 | | 0.527 | | 2,422,816 | | 732,054 | 1,690,762 | |
| Overall | 0.055 | | 0.072 | |  | |  |  | |

^a^Data adopted from Prince et al (Prince et al., 2013).

**Supplementary Table 3**. SNP Heritability partitioned by chromosome 19 and others.

|  |  | Chromosome 19 | |  |  | Others | |  |
| --- | --- | --- | --- | --- | --- | --- | --- | --- |
|  | Heritability | | 95% CI^a^ | | Heritability | | 95% CI | |
| Overall | 11.1% | | 9.4-12.7% | | 15.2% | | 11.4-19.1% | |
| Males | 8.6% | | 5.9-11.4% | | 13.5% | | 4.3-22.8% | |
| Females | 7.8% | | 5.8-9.8% | | 17.5% | | 11.2-23.8% | |

^a^CI: confidence interval

**Supplementary Table 4**. Effect size of *APOE* ε4 in age- and sex-stratified groups.

| *APOE* ε4  alleles |  | | OR (95% CI)^a^ | | | | | | | |  | |
| --- | --- | --- | --- | --- | --- | --- | --- | --- | --- | --- | --- | --- |
|  |  | Male | | | |  |  | Female | | | |  |
|  | Overall | | | 60-80 years | > 80 years | | Overall | | 60-80 years | > 80 years | | |
| 1 | 3.85  (3.40-4.37) | | | 4.26  (3.66-4.96) | 3.06  (2.41-3.88) | | 4.10  (3.71-4.52) | | 4.84  (4.29-5.47) | 2.74  (2.29-3.28) | | |
| 2 | 13.24  (9.79-18.26) | | | 16.05  (11.49-22.96) | 3.61  (1.52-9.05) | | 11.59  (9.19-14.76) | | 14.54  (11.26-19.01) | 3.67  (1.91-7.26) | | |

^a^OR: odds ratio, CI: confidence interval

**Supplementary Table 5**. Effect size of *APOE* ε4 in age- and sex-stratified groups in the randomly selected female sub-cohort with matched numbers of cases and controls as the male cohort.

| *APOE* ε4  alleles |  | | OR (95% CI)^a^ | | | | | | | |  | |
| --- | --- | --- | --- | --- | --- | --- | --- | --- | --- | --- | --- | --- |
|  |  | Male | | | |  |  | Female | | | |  |
|  | Overall | | | 60-80 years | > 80 years | | Overall | | 60-80 years | > 80 years | | |
| 1 | 3.85  (3.40-4.37) | | | 4.26  (3.66-4.96) | 3.06  (2.41-3.88) | | 4.09  (3.63-4.62) | | 4.59  (3.98-5.30) | 2.93  (2.31-3.72) | | |
| 2 | 13.24  (9.79-18.26) | | | 16.05  (11.49-22.96) | 3.61  (1.52-9.05) | | 12.26  (9.14-16.04) | | 14.69  (10.89-20.14) | 3.46  (1.53-8.17) | | |

^a^OR: odds ratio, CI: confidence interval

**Supplementary Table 6**. Significant LD-independent SNPs (*p* < 5×10^-8^) identified in male and female groups using ADGC combined phase 1 and 2 samples. All loci have been reported in previous GWAS.

| SNP | Chr | A1/A2 | Closest Gene | Hetero-  geneity  *p*-value |  | Male | | | |  |  | Female | | | |  |
| --- | --- | --- | --- | --- | --- | --- | --- | --- | --- | --- | --- | --- | --- | --- | --- | --- |
|  |  |  |  |  | Freq | | N | OR (95% CI) | *p-*value | | Freq | | N | OR (95% CI) | *p-*value | |
| **Significant SNPs in males** | | | | | | | | | | | | | | | | |
| rs429358 | 19 | C/T | *APOE* (missense) | 0.067 | 0.226 | | 8060 | 3.245  (2.941-3.579) | 5.04×10^-122^ | | 0.216 | | 11793 | 3.655  (3.370-3.964) | 1.78×10^-215^ | |
| rs440446 | 19 | C/G | *APOE* (intron) | 0.297 | 0.321 | | 7749 | 0.654  (0.604-0.710) | 6.72×10^-25^ | | 0.325 | | 11290 | 0.692  (0.649-0.737) | 1.95×10^-30^ | |
| rs4803764 | 19 | C/T | *NECTIN2* (intron) | 0.643 | 0.279 | | 6773 | 1.466  (1.340-1.603) | 5.55×10^-17^ | | 0.274 | | 9872 | 1.427  (1.329-1.531) | 8.35×10^-23^ | |
| rs7412 | 19 | T/C | *APOE* (missense) | 0.192 | 0.056 | | 8153 | 0.527  (0.449-0.619) | 5.84×10^-15^ | | 0.057 | | 11952 | 0.459  (0.403-0.523) | 1.67×10^-31^ | |
| rs6859 | 19 | A/G | *NECTIN2* (intron) | 0.344 | 0.461 | | 8057 | 1.314  (1.222-1.412) | 1.29×10^-13^ | | 0.459 | | 11605 | 1.374  (1.297-1.456) | 4.87×10^-27^ | |
| rs28399664 | 19 | G/C | *BCAM* (downstream 500B) | 0.913 | 0.016 | | 7859 | 2.568  (1.882-3.503) | 2.67×10^-9^ | | 0.016 | | 11622 | 2.512  (1.969-3.204) | 1.24×10^-13^ | |
| rs138607350 | 19 | G/T | *NECTIN2* (intron) | 0.429 | 0.013 | | 8458 | 2.733  (1.916-3.898) | 2.87×10^-8^ | | 0.011 | | 12454 | 2.277  (1.720-3.013) | 8.71×10^-9^ | |
| rs449647 | 19 | T/A | *APOE* (upstream 2KB) | 0.465 | 0.117 | | 6398 | 0.702  (0.619-0.797) | 4.11×10^-8^ | | 0.119 | | 9443 | 0.661  (0.598-0.731) | 7.31×10^-16^ | |
|  | | | | | | | | | | | | | | | | |
| **Significant SNPs in females** | | | | | | | | | | | | | | | | |
| rs6431220 | 2 | A/G | *LOC105373605* (intron)  near *BIN1* | 0.369 | 0.237 | | 8120 | 0.852  (0.783-0.928) | 2.14×10^-4^ | | 0.240 | | 11887 | 0.811  (0.759-0.867) | 5.03×10^-10^ | |
| rs4748424 | 10 | G/A | *MRC1* (intron) | 0.835 | 0.204 | | 3370 | 0.697  (0.595-0.816) | 6.74×10^-6^ | | 0.210 | | 4949 | 0.682  (0.601-0.774) | 2.52×10^-9^ | |
| rs11541040 | 12 | A/G | *NAP1L1* (intron) | 0.271 | 0.089 | | 8681 | 1.740  (1.421-2.132) | 4.98×10^-5^ | | 0.110 | | 12765 | 2.015  (1.709-2.375) | 3.41×10^-5^ | |
| rs429358 | 19 | C/T | *APOE* (missense) | 0.067 | 0.226 | | 8060 | 3.245  (2.941-3.579) | 5.04×10^-122^ | | 0.216 | | 11793 | 3.655  (3.370-3.964) | 1.78×10^-215^ | |
| rs405509 | 19 | G/A | *APOE* (upstream 2KB) | 0.517 | 0.476 | | 8114 | 0.705  (0.655-0.758) | 7.85×10^-21^ | | 0.478 | | 11821 | 0.683  (0.645-0.724) | 2.67×10^-38^ | |
| rs7412 | 19 | T/C | *APOE* (missense) | 0.192 | 0.056 | | 8153 | 0.527  (0.449-0.619) | 5.84×10^-15^ | | 0.057 | | 11952 | 0.459  (0.403-0.523) | 1.67×10^-31^ | |
| rs6859 | 19 | A/G | *NCTIN2* (intron) | 0.344 | 0.461 | | 8057 | 1.314  (1.222-1.412) | 1.29×10^-13^ | | 0.459 | | 11605 | 1.374  (1.297-1.456) | 4.87×10^-27^ | |
| rs28399637 | 19 | A/G | *BCAM* (intron) | 0.852 | 0.286 | | 5028 | 1.516  (1.368-1.679) | 1.69×10^-15^ | | 0.281 | | 7329 | 1.535  (1.416-1.665) | 3.71×10^-25^ | |
| rs11668327 | 19 | C/G | *TOMM40* (intron) | 0.937 | 0.127 | | 7271 | 0.654  (0.583-0.733) | 4.29×10^-13^ | | 0.127 | | 10535 | 0.650  (0.593-0.712) | 3.96×10^-20^ | |
| rs60049679 | 19 | C/G | *APOC1P1* (upstream 2KB) | 0.572 | 0.034 | | 6372 | 1.868  (1.477-2.362) | 1.85×10^-7^ | | 0.035 | | 9340 | 2.034  (1.702-2.432) | 6.11×10^-15^ | |
| rs28399664 | 19 | G/C | *BCAM* (downstream 500B) | 0.913 | 0.016 | | 7859 | 2.568  (1.882-3.503) | 2.67×10^-9^ | | 0.016 | | 11622 | 2.512  (1.969-3.204) | 1.24×10^-13^ | |
| rs2927468 | 19 | A/G | *NECTIN2* (intron） | 0.523 | 0.444 | | 6022 | 0.758  (0.696-0.285) | 1.58×10^-10^ | | 0.448 | | 8749 | 0.785  (0.734-0.839) | 1.25×10^-12^ | |
| rs8103315 | 19 | A/C | *BCL3* (intron) | 0.147 | 0.119 | | 7835 | 1.210  (1.077-1.361) | 1.38×10^-3^ | | 0.119 | | 11473 | 1.350  (1.233-1.478) | 7.97×10^-11^ | |
| rs2967668 | 19 | G/A | *CBLC* (intron) | 0.693 | 0.090 | | 7089 | 0.701  (0.613-0.802) | 2.06×10^-7^ | | 0.094 | | 10373 | 0.725  (0.653-0.805) | 1.75×10^-9^ | |
| rs111371860 | 19 | T/A | *BCAM-PVRL2* (intergenic) | 0.665 | 0.041 | | 8010 | 0.609  (0.507-0.732) | 1.33×10^-7^ | | 0.040 | | 11758 | 0.642  (0.553-0.745) | 5.53×10^-9^ | |
| rs138607350 | 19 | G/T | *NECTIN2* (intron) | 0.429 | 0.013 | | 8458 | 2.733  (1.916-3.898) | 2.87×10^-8^ | | 0.011 | | 12454 | 2.277  (1.720-3.013) | 8.71×10^-9^ | |
| rs10409808 | 19 | G/C | *APOC4-APOC2 (intron)* | 0.128 | 0.277 | | 1902 | 1.258  (1.064-1.487) | 7.27×10^-3^ | | 0.263 | | 2793 | 1.487  (1.298-1.703) | 1.06×10^-8^ | |
| rs2927439 | 19 | G/A | *BCL3* (downstream 8KB) | 0.998 | 0.321 | | 8176 | 0.839  (0.778-0.906) | 7.50×10^-6^ | | 0.327 | | 12013 | 0.840  (0.790-0.892) | 1.33×10^-8^ | |
| rs74846209 | 19 | T/G | *PPP1R37* (downstream 500B) | 0.847 | 0.103 | | 8676 | 1.319  (1.176-1.478) | 2.12×10^-6^ | | 0.097 | | 12754 | 1.300  (1.185-1.426) | 2.60×10^-8^ | |
| rs79701229 | 19 | A/G | *NECTIN2* (intron) | 0.920 | 0.005 | | 8418 | 3.206  (1.798-5.717) | 7.92×10^-5^ | | 0.005 | | 12425 | 3.326  (2.174-5.090) | 3.08×10^-8^ | |

Abbreviations: Chr: chromosome, A1: effect allele, A2: non-effect allele, Freq: allele frequency of A1, N: sample size, OR: odds ratio, CI: confidence interval*.*

**Supplementary Figure 1.** Manhattan plots of MAGMA-annotated genes in ADGC combined phase 1 and phase 2 samples of the randomly selected subset of female cohort with matched numbers of cases and controls as the male cohort. The red line denotes the gene-based genome-wide significance level of *P* value = 2.61×10^-6^.

**
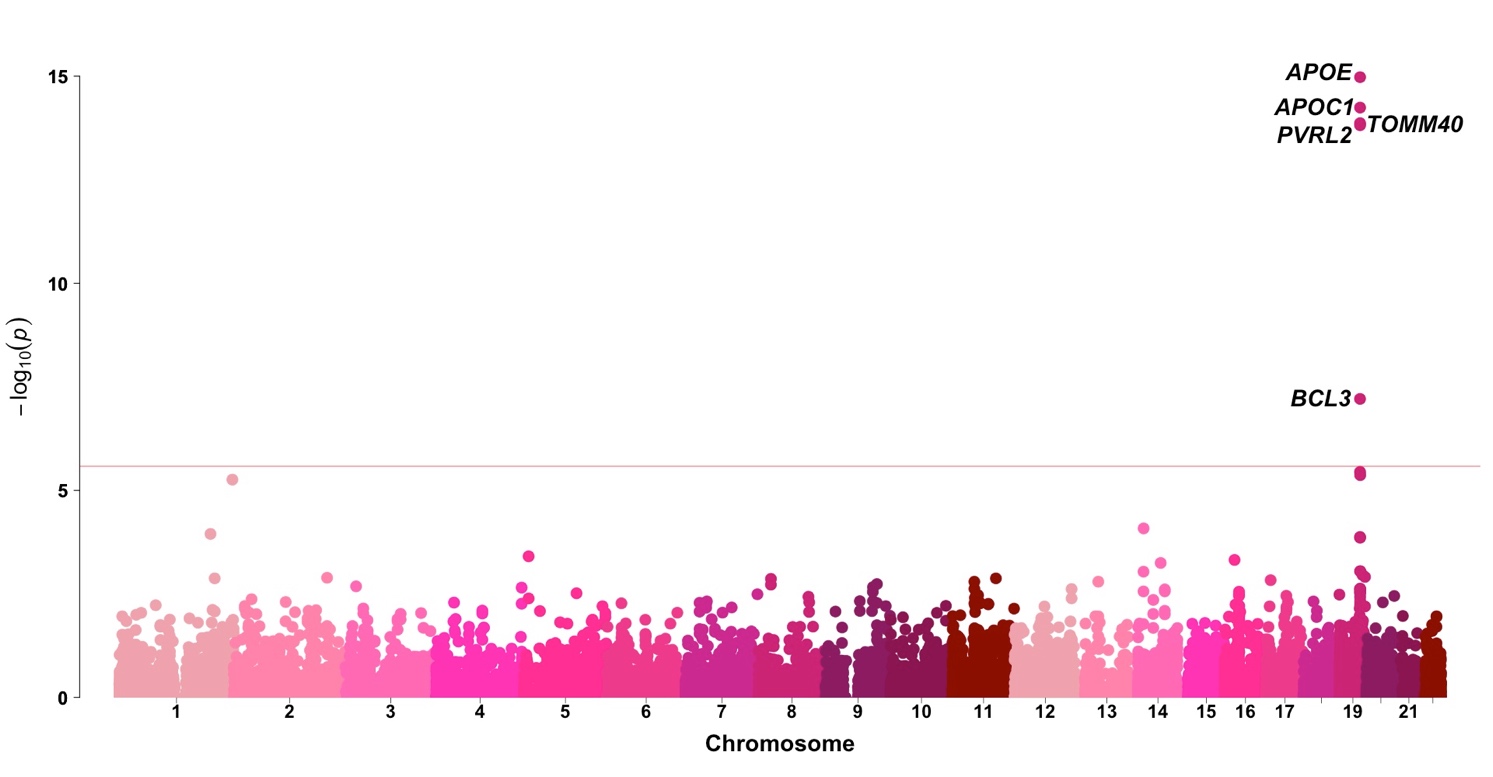
**

**Supplemental Table 7.** Significant MAGMA-annotated genes (*P* value < 2.61×10^-6^) using GWAS on the female sub-cohort with matched numbers of cases and controls as the male cohort. The top SNPs with smallest *p*-values within genes are shown.

| Gene | Chr | *p-*value | Top SNP in Gene | A1/A2 |  | Male | | | |  | | Matched Female Sub-cohort | | | |  | Heterogeneity between sexes |
| --- | --- | --- | --- | --- | --- | --- | --- | --- | --- | --- | --- | --- | --- | --- | --- | --- | --- |
|  |  |  |  |  | Freq | | N | OR  (95% CI) | *p-*value | | Freq | | N | OR  (95% CI) | *p-*value | |  |
| **Top genes in matched female sub-cohort** | | | | | | | | | | | | | | | | | |
| *APOC1* | 19 | 5.72×10^-15^ | rs12721051 | G/C | 0.755 | | 7374 | 2.80  (2.70-2.90) | 9.71×10^-97^ | | 0.768 | | 7459 | 3.27  (3.17-3.36) | 8.71×10^-129^ | | n.s. |
| *APOE* | 19 | 1.05×10^-15^ | rs429358 | C/T | 0.774 | | 8060 | 3.25  (3.15-3.34) | 5.04×10^-122^ | | 0.792 | | 8029 | 3.73  (3.63-3.83) | 1.27×10^-148^ | | n.s. |
| *TOMM40* | 19 | 1.53×10^-14^ | rs59007384 | T/G | 0.713 | | 7699 | 2.42  (2.33-2.51) | 7.85×10^-85^ | | 0.730 | | 7642 | 2.58  (2.50-2.67) | 1.21×10^-99^ | | n.s. |
| *PVRL2* | 19 | 1.36×10^-14^ | rs6857 | T/C | 0.762 | | 7955 | 2.81  (2.71-2.90) | 4.65×10^-101^ | | 0.777 | | 7890 | 3.06  (2.97-3.15) | 2.25×10^-120^ | | n.s. |
| *BCL3* | 19 | 6.19×10^-8^ | rs2965169 | C/A | 0.610 | | 6932 | 0.81  (0.73-0.90) | 5.80×10^-7^ | | 0.598 | | 10174 | 0.82  (0.74-0.89) | 1.85×10^-7^ | | n.s. |

Abbreviations: Chr: chromosome, A1: effect allele, A2: non-effect allele, Freq: allele frequency of A1, N: sample size, OR: odds ratio, CI: confidence interval, n.s.: not significant.

**References**

Jun, G., Ibrahim-Verbaas, C. A., Vronskaya, M., Lambert, J.-C., Chung, J., Naj, A. C., et al. (2016). A novel Alzheimer disease locus located near the gene encoding tau protein. *Mol. Psychiatry* 21, 108–117. doi:10.1038/mp.2015.23.

Jun, G., Naj, A. C., Beecham, G. W., Wang, L.-S., Buros, J., Gallins, P. J., et al. (2010). Meta-analysis confirms CR1, CLU, and PICALM as alzheimer disease risk loci and reveals interactions with APOE genotypes. *Arch. Neurol.* 67, 1473–1484. doi:10.1001/archneurol.2010.201.

Naj, A. C., Jun, G., Beecham, G. W., Wang, L.-S., Vardarajan, B. N., Buros, J., et al. (2011). Common variants at MS4A4/MS4A6E, CD2AP, CD33 and EPHA1 are associated with late-onset Alzheimer’s disease. *Nat. Genet.* 43, 436–441. doi:10.1038/ng.801.

Prince, M., Bryce, R., Albanese, E., Wimo, A., Ribeiro, W., and Ferri, C. P. (2013). The global prevalence of dementia: a systematic review and metaanalysis. *Alzheimers. Dement.* 9, 63-75.e2. doi:10.1016/j.jalz.2012.11.007.
